# The role of the National Health Insurance Scheme in strengthening delivery of health services among the elderly at district level in Zambia: a qualitative case study

**DOI:** 10.1186/s12889-026-26390-9

**Published:** 2026-01-31

**Authors:** Lucky Sakala, Cosmas Zyambo, Malizgani Paul Chavula

**Affiliations:** 1 Department of Community and Family Medicine, School of Public Health, University of Zambia, Ridgeway Campus, Box 50110, Lusaka, Zambia; 2 Department of Epidemiology and Global Health, Faculty of Medicine, Umeå University, Umeå, 901 87 Sweden

**Keywords:** Health insurance, Health financing, Challenges, The elderly

## Abstract

**Background:**

In recent years, the international health discourse has increasingly focused on achieving universal health coverage (UHC) through the provision of health insurance. Low- and middle-income countries (LMICs), such as Zambia, have adopted national health insurance schemes to improve health service delivery among their citizens. This study explored the role of Zambia’s National Health Insurance Scheme (NHIS) in strengthening the delivery of health services to the elderly at the district level in Zambia.

**Methods:**

The study employed a qualitative case approach, and purposive sampling was used to select 22 participants for in-depth and key informant interviews. These included 14 elderly NHIS members, 6 staff members from health insurance facilities, and 2 NHIMA provincial office managers. The data was analysed using a thematic analysis approach. This was achieved by identifying patterns in service delivery and user experiences in accessing health services.

**Results:**

The study findings revealed that the National Health Insurance Scheme (NHIS) has improved healthcare access for the elderly by offering affordable and increasing utilisation of services through accrediting facilities and reducing financial risks. Outreach efforts have helped register elderly members directly from their homes. However, challenges remain, including medication shortages, inadequate healthcare providers, long-distance travel, and system inefficiencies. The strategies to address the challenges include the government increasing medical subsidies, improving monitoring of the NHIS fund, policy reform to enhance equitable elderly care, and health infrastructure expansion.

**Conclusion:**

While NHIS might have enhanced health service access among the elderly, service-related gaps still hinder its optimal effect. The challenges include limited health facility accreditation, shortages of medicines, and weak governance. Overcoming the barriers requires ethical NHIS reforms that promote better accreditation of healthcare, increased funding for improved infrastructure, and universal availability of medical services.

**Supplementary Information:**

The online version contains supplementary material available at 10.1186/s12889-026-26390-9.

## Introduction

In 2022, the number of elderly individuals aged 60 years and above in sub-Saharan Africa was estimated to be around 78 million [1]. Many of these older people face a dual burden of both communicable and non-communicable diseases, such as diabetes, hypertension, musculoskeletal disorders, and mental health conditions, as evidenced among older people in many African care homes [2]. The elderly in Africa are burdened with precarious socioeconomic circumstances, including shrinking income-generating capacities and diminished informal support networks. These limit their ability to pay for care or travel long distances to district-level facilities. To address these challenges, several African countries are actively reforming their health systems by introducing national health insurance schemes and strengthening primary healthcare systems, as key strategies to achieve Universal Health Coverage (UHC). This is to ensure equitable access to quality health for all, including the elderly, while reducing out-of-pocket expenditure and financial vulnerability. This approach has been adopted as part of the broader health financing reforms aimed at improving service delivery and social protection [3]. In recent years, international efforts have prioritised achieving UHC through health insurance. In 2005, the World Health Organisation (WHO) passed a resolution at the 58th World Health Assembly encouraging countries to adopt UHC within their health systems [4]. The resolution promoted social health insurance as a means to mobilising additional resources for healthcare, risk pooling, expanding access to healthcare for the vulnerable, and improving the quality of healthcare, particularly in low-income countries (LIMCS) [5].

Many low- and middle-income countries (LMICs) have explored ways to introduce and expand health insurance coverage as a tool for health financing. They aimed to implement effective exemption mechanisms for those unable to pay for their health expenses by increasing tax collection and reallocating funds towards universal healthcare financing for all [6]. However, most LMICs struggle to adopt health insurance models due to the resources needed to run an effective National Health Insurance Service (NHIS). Limited government revenue and funding make it difficult to subsidise healthcare costs, especially high-cost services for the poor and elderly [7].

NHIS plays a vital role in enhancing equitable access to quality health services, particularly for vulnerable groups such as the elderly. Since elderly people are prone to chronic conditions like hypertension, diabetes, and cardiovascular diseases, NHIS provides a financial protection mechanism that reduces out-of-pocket expenditures, preventing catastrophic health costs and poverty among the elderly [8]. Moreover, it facilitates access to comprehensive and continuous care by pooling resources to ensure the availability of essential medicines, diagnostic services, and age-friendly healthcare at the district level. For Zambia, the NHIS is crucial in promoting UHC, strengthening health service delivery, and improving health outcomes for older citizens through sustainable and inclusive financing [9].

In 2018, Zambia introduced the National Health Insurance Management Authority (NHIMA) to strengthen healthcare financing and improve access, particularly for vulnerable groups, such as the elderly. The Authority was established under the NHIS Act No. 2 of 2018, and began its operations in 2019. It was realigned under the Ministry of Health (MoH) in 2024 to enhance NHIMA operations through policy implementation, coordination, and governance [10]. NHIMA plays a crucial role in the daily management of the scheme. This includes overseeing the insurance fund, accrediting providers, registering members, and processing claims. It offers a wide range of services—including outpatient and inpatient care, diagnostics, surgeries, and chronic disease management—without point-of-care charges. While formally employed individuals contribute 2 per cent of their basic income, informal sector households pay a monthly fee. Children and seniors aged 65 and above are exempt from contributions [11]. Furthermore, the insurance scheme enables universal access to health services, including elderly care. In this regard, the Authority has expanded healthcare access for the elderly by reducing financial barriers and increasing service availability [12]. This has contributed to an increase in registered members from 1.6 million in 2021 to over 4.6 million by mid-2024, representing approximately 23 per cent of the population [8]. Currently, the elderly also receive preventive, curative, and palliative care from selected public and private providers. For instance, access to healthcare has increased in rural districts such as Chirundu district, where outpatient visits rose from 67,048 in 2019 to 74,686 by mid-2022 [13]. This suggests that financial protection is encouraging healthcare use among older adults in Zambia. However, challenges remain in rural areas where there is often a lack of accredited facilities, members face delays in the claim processing, there are shortages of essential medicines, and difficulties navigating the system [14].

Therefore, NHIMA should expand its provider network in underserved areas, streamline administrative processes, and prioritise geriatric care [9]. Training healthcare workers in elderly care and ensuring access to medications for chronic diseases can also improve health outcomes and access to NHIMA services. Trained workers can also leverage digital health tools as outlined in Zambia’s National Health Strategic Plan 2022–2026, and reduce travel burdens, thereby enhancing service delivery [15]. Despite NHIMA’s progress in delivering healthcare services to all, including the elderly, there is still limited research on its impact among the elderly in Zambia. Future studies should investigate how the scheme impacts health outcomes in districts like Kitwe, informing policies that address the complex needs of aging populations [9].

Furthermore, studies from Tanzania highlight the importance of age-sensitive evaluation in health insurance schemes. However, even there, while insurance has improved healthcare utilisation among the elderly in rural areas, challenges persist around responsiveness, equity, and access [16]. Zambian studies have also shown that NHIS has expanded coverage and reduced out-of-pocket costs, yet elderly members still face barriers such as limited access in rural areas, shortages of essential medicines, and administrative hurdles [17]. The findings of this study underscore the need to assess how NHIS addresses the complex needs of the aging population, particularly in the underserved areas of Kitwe district in Zambia. Despite Zambia’s progress, research on NHIS impact among the elderly remains scarce, with most studies focusing on general healthcare access [13]. Therefore, this study aimed at exploring the role of the national health insurance scheme in strengthening health services delivery among the elderly at district level in Zambia.

## Methods

### Study setting

This study was conducted in Kitwe District Zambia. It is Zambia’s second-largest city, located in the Copperbelt Province, with a population of about 823,625 [18]. The major economic activities in this city are mining, manufacturing, informal trade, and small-scale farming, which shape healthcare access. Formal workers often benefit from structured coverage, while informal workers face financial and logistical barriers. The study included NHIS members aged 55 and above, reflecting Zambia’s retirement framework and capturing views from retirees and active contributors under the National Pensions Scheme Amendment Act No.7 of 2015 [3]. NHIMA, an institution created under the 2018 Act, runs Zambia’s mandatory, income-based insurance scheme and has accredited over 400 facilities as of 2024 [10]. The research selected five NHIMA-accredited facilities to represent both public and private health providers as participants in the study.

### Study design

This study employed a qualitative case study design to investigate how the NHIS facilitates healthcare access for the elderly in Kitwe District. This approach enabled the researchers to gain an in-depth understanding of the experiences of elderly members, including the benefits they receive and the challenges they face when accessing NHIS-accredited services. The study also gathered insights from healthcare providers, NHIS administrators, and other stakeholders to identify potential solutions. Case study designs are particularly useful for examining complex social phenomena within real-life contexts, making them well-suited for health systems research [19].

### Data collection methods

Data was collected through in-depth interviews with 22 participants, selected using purposive sampling. This was to ensure relevance to the study objectives. The sample included 14 elderly NHIS members, 6 NHIS health facility representatives, and 2 NHIMA managers from the provincial office. The elderly aged 55 and above, both male and female, were interviewed, in line with Zambia’s retirement framework. All the key informants were stakeholders directly involved in NHIS service delivery. This approach allowed the researchers to gather diverse perspectives from both service users and implementers. Each interview lasted approximately 30 min and was conducted after obtaining informed consent. The interviews were designed to explore participants’ experiences, challenges, and perceptions of NHIS’s role in elderly healthcare. A structured guide for key informants to obtain information on their roles and insights into service delivery was developed. Interviews were conducted until the study reached thematic saturation. Accuracy was ensured by listening to audio interviews against reading all the transcripts. All data was securely stored on a password-protected computer to maintain confidentiality (Table 1).

**Table 1:** Participants, categories, and description

Interview Type	Category	Number of Participants	Description
IDIs	Elderly male NHIS members	7	Elderly male NHIS members 55years and above
IDIs	Elderly female NHIS members	7	Elderly female NHIS members 55years and above
KIIs	Health facility staff	4	Nurses, midwives, and other frontline health
KIIs	Health insurance facility staff	2	NHIS customer service personnel
KIIs	Health insurance staff	2	Personnel involved in managing or implementing health insurance schemes
Totals		22	

### Data analysis

The data was analysed using thematic analysis, following the six-step process outlined by Braun and Clarke [20]. The first step involved familiarisation of the collected data. The researcher read and listened to the transcripts and audio recordings multiple times to get an in-depth understanding of the data, then generated initial codes by identifying recurring ideas and patterns across interviews. These codes were grouped into sub-themes, which we reviewed collaboratively to ensure they accurately reflected the data and remained internally consistent. After refining and naming each theme, findings were interpreted in relation to the study’s aim, which was to understand how NHIS influences healthcare access and delivery for the elderly.

### Findings

This study explored the role of the national health insurance scheme in strengthening health services among the elderly. The findings are organised as follows: facilitators of the elderly in accessing the services, barriers for the elderly in accessing health services and suggested strategies to improve access to health services among the elderly (Table 2).

**Table 2:** Summary of themes, facilitators, barriers, and strategies influencing elderly access to NHIS-accredited services

Main Themes	Sub-Themes
Theme 1: Facilitators of the elderly in accessing the services	a) Provision of affordable health care b) Accreditation of health facilities c) Provision of financial risk protection d) Awareness and registration of elderly members from homes
Theme 2: Barriers for the elderly in accessing health services	a) Lack of medications at NHIS facilities b) Long distance to accredited facilities c) System failure and delayed approval requests
Theme 3: Suggested strategies to improve access to health services among the elderly	a) Introduction of government subsidy on NHIS b) Monitoring the utilisation of NHIS funds by the government c) Construction and accreditation of more NHIS facilities near the elderly

### Theme 1: Facilitators that influence the elderly in accessing NHIMA services

Facilitators that influenced the elderly to access NHIS services included the provision of affordable healthcare, accreditation of health facilities, provision of financial risk protection, and awareness and registration of elderly members from their homes.

#### Provision of an affordable health insurance scheme for the elderly

Throughout the interviews, elderly NHIS members indicated that the scheme has provided affordable health services for them. They recognised positive outcomes of the NHIS, and these included reduced out-of-pocket expenses. For example, it was found that those on the senior citizens’ account and those on the pensioners’ accounts do not make any payments to the scheme, allowing them to enjoy their retirement money without spending it on health expenses. However, participants aged 55 to 64 years old are subjected to an income assessment to determine how much their premium [21].

“*If possible*,* paying more is better to assist even those who can’t afford it. Health is costly; people need to invest and pay more for health services while they are still working so that they enjoy free services when they are old. Like me*,* who’s not making any payments.” (An 80-year-old male, NHIS member, 01 IDI)*

*“After the assessment*,* the NHIMA office told me to contribute K30 monthly*,* which is affordable*,* and I can access the same services as those paying more than K30.” (55-year-old female, NHIS member,02 KII)*

#### Accreditation of various government and private facilities near the people

The interviewee at the NHIMA office indicated that the health insurance authority has embarked on accreditation of various private and government facilities that meet the required standards to ensure quality and achieve universal health services for all in Zambia [12]. Additionally, the authority has also included several private health facilities. Although NHIMA has accredited various private and government facilities, participants expressed concern about the NHIS being located mostly in central town rather than in rural parts of Kitwe, where most of the elderly members reside.

“*For rural areas*,* the problem we have is that facilities do not meet our requirements. Most of them are low-standard talk in places like Kamfinsa and Kitwe West. Most private facilities there are of low standards*,* so we end up accrediting the district hospital instead. As for now*,* we are accrediting the district*,* mission*,* and private clinics that have equipment that can help people before they are referred to big hospitals*,* like the general and the teaching hospitals.” (Government Official, 03 KII)*

#### Provision of financial risk protection

Many elderly paying participants indicated that they contribute to the scheme every month to secure financial protection against future catastrophic health expenditures that they might not be able to afford. They also indicated that although the funds may not be used immediately, advance payments allowed them to access health services without the burden of future medical bills [11]. This proactive approach provides peace of mind, knowing that their future healthcare needs are already covered.

“*I pay K30 to NHIMA and have access to services when I visit a health facility. I love to pay six months in advance to avoid being inconvenienced when sickness and I have added my children and grandchildren to NHIMA.’’ (57-year-old female, NHIS member, 04 IDI)*

#### Awareness and enrolment of elderly members from home

Findings reveal that NHIMA agents are everywhere across the country, moving door-to-door, church to church, to register the elderly people to the scheme. Respondents indicated that officers from the health scheme followed them to their respective homes and trading places to register them. A NHIMA staff member indicated that the introduction of the door-to-door campaign was to follow people to their homes so that no one would be left behind. Outreach campaigns have been launched to target those in remote areas that are far from NHIMA provincial offices.

*“Informal registrations for the informal sector are ongoing*,* and we have officers on the ground in different towns. In addition*,* we are also conducting online registration where you need a tablet to capture the NRC*,* and the member will be registered there*,* and then. Through our trips*,* we visit various towns for informal sector registration. Services are found at various district hospitals*,* and we plan to accredit more facilities to help with the huge numbers.” (Government Official, 05 KII)*

*“Last year*,* officers from NHIMA followed us in the communities to register us as the elderly and told us it’s free of charge*,* no payments. So*,* I went with my wife’s NRC at the hospital so that they also register my wife under my account.” (A 67-year-old male, NHIS member, 06 IDI)*

### Theme 2: Barriers the elderly face in accessing health services

According to the study participants, the barriers to accessing healthcare services faced by the elderly included medication shortages at NHIS-accredited facilities, long distances to these facilities, system failures, and delays in approving requests.

#### Lack of medication at NHIS hospitals and pharmacies near the members

Participants revealed that one of the barriers to accessing health services at NHIS-accredited facilities, including both private and government facilities, was a lack of medication at the pharmacy. The participants indicated that the Ministry of Health was grappling with a significant challenge of medicine shortages across its facilities nationwide. As a solution to this problem, NHIMA has accredited several private pharmacies to facilitate members’ access to medicines. Despite this effort, NHIS members continue to face difficulties accessing medicines at nearby facilities, often being informed of stock-outs and sometimes, directed to purchase essential medications out-of-pocket. This has adversely affected health outcomes, particularly for elderly individuals who find it financially burdensome to procure necessary medicines for their geriatric care.

*“No medicines at hospitals and pharmacies*,* NHIMA needs to find a solution. We walk to hospitals and stand in long queues only to be told no medicine. This is a challenge for us elderly. Other services like huge scans are only in Lusaka*,* meaning I need transport to go that side*,* which is too costly for me*.” *(A 62-year-old female, NHIS member, 07 IDI)*

#### Long distance to the accredited NHIS facilities for the elderly in the rural areas

The participants in rural parts of Kitwe revealed that covering long distances to access NHIS services from private and public facilities led to accessibility barriers and transportation costs. Most of the clinics that are near people are not under NHIS. Instead, the elderly participants need to go to a government or private hospital to access the services. Participants in areas such as Kamfinsa and Kitwe West travel to Kitwe Central Hospital to access quality medical services once they are referred from the clinics.

“*Other services like huge scans are only at the central hospital*,* meaning I need transport to go that side*,* which is too costly for me. Transport to the pharmacy*,* especially those that are far away*,* and sometimes when the pharmacy does not have*,* you are forced to buy.” (A 66yrs old female, NHIS member, 08 IDI)*

#### System failure and delayed approval requests

The study participants revealed that system failures pose a significant challenge for NHIMA members. NHIS staff are unable to log in and verify a client’s status, rendering them unable to provide services. Typically, members visiting a NHIMA desk are required to present their NRC or membership card, which the staff use to log in and check the member’s account profile and status before delivering services. However, system failures hinder access to services for both paying and non-paying members. Furthermore, participants stated that certain services, such as operations, scans, and optical services, require prior authorisation from NHIMA, which can take at least 48 h to approve. This delay poses a significant challenge for emergency cases that require immediate attention.

*The system is very slow. Sometimes*,* they take time looking for our records*,* and the record-keeping is very poor. Let those in public facilities learn how to keep records like private ones.” (77-year-old male, NHIS member, 09 IDI)*

*“There was a time I wanted to get spectacles*,* and I faced challenges. Central hospital told me to wait for two weeks*,* but it took 3 months to get the glasses.’’ (58-year-old female, NHIS member, 10 IDI)*

### Theme 3: Suggested strategies to improve access to health services among the elderly

According to participants, suggested strategies to improve access to healthcare services among the elderly included introducing a government subsidy to support contributions for unemployed NHIS elderly members, enhancing government oversight and monitoring of NHIS fund utilisation, and constructing and accrediting more NHIS facilities near elderly populations.

#### Introduction of a government subsidy to help with contributions for unemployed NHIS elderly members

According to the respondents, all elderly members between 55 and 64 years old and non-retired from the formal sector, must pay a monthly subscription fee to remain active on the scheme. The NHIS scheme was designed to allow only retirees from the formal sector and those 65 years and above to be exempted from payments; the rest of the members, 18 to 64 years, must contribute to the scheme monthly. To increase affordability, elderly members indicated that the government must introduce a subsidy that can sustain the accounts of elderly members from 55 to 64 years, for example, a contribution waiver, as most of them face economic challenges and cannot afford to pay their NHIS monthly premiums.

“*The government needs to provide a fund specifically for non-paying members that can help with their medical bills. Some elderly people who are 60years cannot afford to pay 30 or 60 kwacha every month government needs to come in and help the poor.” (NHIS staff, 12 KII)*

#### Monitoring the utilisation of NHIS funds by the government

The interviewees also reported that monitoring NHIS funds is crucial for ensuring transparency and accountability. Effective monitoring is also essential for improving service delivery, preventing misappropriation of funds, and curbing corrupt practices. The Ministry of Health can play a vital role in overseeing the fund and ensuring that it is utilised for healthcare services that cater to the needs of both paying and non-paying NHIS members.

*“The government can help us with more funding and monitor how we are utilising the resources so that everyone benefits from this health scheme. This will help to sustain the fund so that the scheme does not collapse.” (Government Official, 13 KII)*

#### Construction and accreditation of more NHIS facilities closer to the elderly

The participants indicated a need to construct and accredit more health facilities under NHIS closer to the people to overcome the challenges of long distances and congestion at the facilities. NHIMA has a few facilities to service its increasing membership, which has caused a lot of congestion and long queues at the facilities. Building and accrediting more NHIS facilities, such as pharmacies in areas such as Kamfinsa and Kitwe West, will help the elderly with the cost of travelling to the town centre when they need NHIS services.

*The government needs to build more facilities for NHIMA to accredit. We need more clinics under NHIMA. We are now a lot under NHIS*,* so even the facilities and NHIS need to be increased so that people have many options.” (Government Official, 15 KII)*

## Discussion

This study explored the role of the National Health Insurance Scheme (NHIS) in enhancing healthcare services for the elderly. The key findings indicated that NHIS has enhanced access to affordable healthcare by accrediting both public and private facilities, offering financial risk protection, and facilitating registration of elderly members at their homes. Despite these benefits, the study identified challenges, such as medicine shortages at hospitals and NHIS-accredited pharmacies, lengthy queues at facilities, and significant distances to facilities, particularly in rural areas of Kitwe. To address these issues, the study recommends expanding the accreditation of more NHIS facilities closer to the members, ensuring the availability of medicines, and strengthening government oversight of the NHIS fund management.

### The role of NHIS in strengthening health services among the elderly

This study’s findings suggest that the National Health Insurance Scheme (NHIS) plays a vital role in providing affordable healthcare coverage to elderly members. From the perspectives of the participants, NHIS has become an enabler of healthcare access and utilisation by the elderly. It eliminates out-of-pocket payments, which were a prerequisite for access to health care before the introduction of NHIS [16]. This finding aligns with the provision of affordable healthcare for the elderly, which protects them from incurring high costs associated with access to healthcare services [8]. It shows an increase in the utilisation of health services among NHIS elderly active members for both inpatient and outpatient care, compared to those not under NHIS. The finding was consistent with a study done in Zambia that confirmed that public health insurance schemes can enhance affordability, access, utilisation, financial protection, and overall health status, ultimately contributing to universal health coverage for elderly citizens who lack a steady income to cover medical bills [16].

Further, the findings from this research have shown that NHIS has the potential to provide risk protection by reducing the burden of healthcare costs among the elderly, making it a key mechanism for achieving universal health coverage [22]. Due to this financial protection, participants acknowledged HI as a promoter of equitable access based on needs, not social status [23]. To support this finding, a study was carried out in Zambia, which pointed out that all active NHIS members have equal access to health services regardless of their social or economic status. The study further showed that elderly people with HI were able to use outpatient and inpatient healthcare, like any other NHIS member who was contributing more to the scheme [13]. This finding is not similar to NHIS findings in countries like Ghana, where inequitable access was evident, as those who pay more, for example, the rich, will have better access to health services than the poor and vulnerable [16].

Furthermore, the study findings also indicate that the NHIS plays a significant role in strengthening healthcare services for the elderly by accrediting both private and public facilities. As the number of elderly members registered on NHIS continues to grow, there is a pressing need to accredit more facilities, both in urban and rural areas across the country [11]. Participants stressed the need for more facilities near them to increase access to health services. This is in line with a study done in Tanzania, which suggested that one of NHIS’s key objectives is to increase access to healthcare services by increasing the number of accredited facilities that can help alleviate congestion and long queues found at most facilities. By expanding the network of accredited facilities, elderly members will be able to access NHIS services at facilities of their choice without prolonged waiting times [21].

#### Barriers for the elderly in accessing health services

The study also highlighted the challenges hindering the NHIS from strengthening the quality of health services among its elderly members. The participants reported challenges, including shortages of essential medicines and a lack of skilled human resources at most facilities. A study by the Policy Monitoring and Research Centre (PMRC) confirmed that most government hospitals and accredited NHIS pharmacies experience shortages of drugs and skilled human resources trained to care for the elderly members [11]. This finding is consistent with studies done in Zambia and Nigeria that pointed out that healthcare facilities in rural areas of Zambia experience a lack of medicines due to delayed deliveries from the central medical store department in urban areas. This shortage of essential medicines needed by the elderly at NHIS facilities forces the insured to pay for drugs and laboratory services, despite being under NHIS, for them to have access to free health services, on time [8, 24]. 

In addition , the participants also indicated that system failure was another barrier to accessing health services under NHIS. Most service providers interviewed revealed that they cannot attend to any client once the system is down. In addition, they are unable to bill or process any claims, leading to delayed reimbursements [17]. This finding aligns with a study done in Tanzania, which showed that delayed reimbursement can highly affect service delivery. Some facilities might not be completing claims on time due to a lack of effective systems, difficulties in understanding of the process, and bureaucratic procedures [2]. To support this finding, a study done in Kenya confirmed that inefficiencies due to wastage, misappropriation, bureaucracy, inefficiency claims and fraud within the health system caused major hurdles for universal health coverage financing [25]. 

Furthermore, the elderly participants pointed out that challenges far beyond logistical or financial issues strike at the heart of ethical healthcare delivery. This is in line with a study done in Ghana that showed equity of access demands that the elderly are not just included in policy but prioritised, given their vulnerabilities [26]. For example, participants in the Kamfinsa constituency stated that they reside far from the accredited NHIS facilities located in the central town. This geographical barrier limits their access to NHIS facilities, as most of them face transport cost challenges [7]. This finding concurs with a study done in Tanzania where elderly participants complained that NHIS facilities under NHIS are far from their location, hence, they are forced to spend on transport for them to have access to NHIS services [16].

### Suggested strategies to improve access to health services among the elderly

From the perspective of the participants, increasing awareness and education of NHIS among the elderly is one of the strategies to increase access and utilisation of health services among the elderly. In line with the policy monitoring and research centre, PMRC, many elderly individuals may not fully understand how to access or benefit from NHIS. Both the government and health authorities should implement targeted education campaigns through community health workers, local media, and social groups to improve awareness of NHIS services, especially tailored for the elderly, on the benefit package [12]. Other participants stressed the need to train health workers at the facilities on geriatrics care, as most of them complained about experiencing mistreatment. A study done in Nigeria suggested that healthcare providers should receive training focused on elderly care within the NHIS framework to improve service delivery. This includes understanding age-related conditions and how to communicate effectively with the elderly patients under NHIS [24].

Nevertheless, the participants also pointed out the need to strengthen infrastructure and service provision to improve access and utilisation. NHIS coverage should be matched by the availability of quality services. Investment is needed to ensure that essential medicines, diagnostic services, and qualified personnel are available in public and private facilities used by the elderly under NHIS [27]. Participants suggested that the availability of health insurance infrastructure that is near to the people, especially the elderly, can be a good solution in addressing the challenges of long distances in accessing quality health services by the elderly [17]. This was strongly supported by a ministerial directive from the Zambian minister in 2024, stating that NHIMA accreditation must not be restricted to only district and higher-level hospitals, but zonal health centres including mini hospitals. By so doing, this will bring NHIS services closer to the people [28]. Furthermore, there is a need for improvements in system failures and access procedures. Accessing services through NHIS can be challenging for older adults, especially when the system is down. Simplifying procedures, reducing paperwork, and offering support at health facilities can help service providers better navigate the system and be able to offer services [29]. 

In addition, the service providers suggested monitoring and evaluation of NHIS funds by the government to make sure that the funds are utilised for the intended purpose. The government of Zambia under the Ministry of Health gave NHIMA a mandate to manage the NHIS fund through an act of Parliament No. 2 of 2018 [10]. A Zambian study suggested that there is need to establish regular monitoring and evaluation mechanisms specifically focused on elderly beneficiaries. Feedback from elderly users should be incorporated into policy revisions to ensure that the NHIS remains responsive to their needs [5]. In addition, a study done by the World Bank on overcoming some challenges, concluded that policy efforts should focus on adjusting social health insurance and optimising healthcare resource allocation. This is to enhance the effective utilisation of healthcare services and control cost increases among middle-aged and elderly adults. With a policy in place, resources will be channelled to the right purpose, to keep the scheme afloat [4].

### Limitations of the study

This study was limited by its qualitative design, which, while effective for gaining in-depth insights, restricts the generalisability of the findings to the wider elderly population in Zambia. The research was conducted in Kitwe District alone. Therefore, regional variations in the implementation and impact of the NHIS may not be reflected. Additionally, the study relied on self-reported data from participants, which may be subject to recall bias or social desirability bias. Language barriers and differences in literacy levels among some elderly participants may have also influenced the clarity and depth of responses. Lastly, due to time and resource constraints, the sample size was relatively small, potentially limiting the diversity of perspectives captured.

## Conclusion

This study explored the role of the national health insurance scheme in strengthening health services among the elderly in the Kitwe district. The findings indicate that NHIS provides a strong policy foundation for improving health service delivery to the elderly. However, without ethical, equity-focused reforms, especially targeting delays in reimbursements, enhancing benefit packages, and strengthening governance, its impact will be diminished [6]. The study also found that NHIS in Zambia is experiencing several challenges, such as the lack of medications in most accredited NHIS facilities, especially public facilities, long queues, and congestion at the NHIS facilities. Members spend long hours waiting for them to be attended to. The long distance to facilities in most rural parts of Kitwe is another challenge, as elderly members must travel long distances to access better services. Moreover, the study also explored potential solutions to enhance NHIS services for the elderly. One proposed solution was the introduction of a government subsidy to support elderly members in paying their premiums, enabling them to remain active and access healthcare services. Additionally, the study recommends that government oversight and monitoring of NHIS funds be enhanced to promote transparency and accountability. Overall, the NHIS plays a vital role in strengthening healthcare services for its members and advancing universal health coverage.

## Supplementary Information

Supplementary Material 1.

## Data Availability

The datasets used and/or analysed during the current study are available from the corresponding author on reasonable request.

## Abbreviations

NHIS: National health insurance scheme
UHC: Universal Health Coverage
LMIC: Low- and Middle-Income Countries
WHO: World Health Organisation
HI: Health Insurance
NHIMA: National Health Insurance Management Authority
