# Supplementary material for: The role of the National Health Insurance Scheme in strengthening delivery of health services among the elderly at district level in Zambia: a qualitative case study
Source: BMC Public Health. 2026 Jan 31;26:725. doi: 10.1186/s12889-026-26390-9 (PMC12930869; doi:10.1186/s12889-026-26390-9)
Supplement: Supplementary file 1 — Supplementary Material 1. [file 12889_2026_26390_MOESM1_ESM.docx]

**Study Title: TO EXPLORE THE ROLE OF THE NATIONAL HEALTH INSURANCE SCHEME IN STRENGTHENING HEALTH SERVICES AMONG THE ELDERLY IN KITWE DISTRICT.**

**Interview ID: 01**

**Interview Date: 6^th^ MAY 2024**

**Number of Participants: 01**

**Participant Title: NHIMA ELDERLY PRINCIPAL MEMBER (Retiree)**

**Site: Kitwe District**

**Transcriptionist: SAKALA LUCKY**

**DEMOGRAPHIC CHARACTERISTICS OF RESPONDENTS**

**I: Gender?**

R: Male

**I: How old are you sir?**

R: according to years on my NRC I am a 69years old Zambia senior citizen

**I: What are you currently doing sir your occupation?**

R: I used to work in the mine back then but am now retired just helping my wife with home gardening and house chores.

**I: Are you married Sir is mum around?**

R: Yes, am married with 5 Children and a lot of grandchildren.

**I: how far have you going with your education sir?**

R: I reached form IV that senior secondary level

**SECTION B.**

**ROLE OF THE NATIONAL HEALTH INSURANCE SCHEME IN STRENTHENING SERVICES DELIVELY AMONG THE ELDERLY**

**I: As a member of NHIMA member Sir do you access health care/ seek treatment from NHIMA hospital anytime you are sick without facing any difficulties, If the answer is No what challenges do you face?**

R: NO not every time some time I face challenges when I visit the hospital especially government NHIMA facilities. For example, there are a lot of queues one needs to go the hospital as early as 06hrs. Sometimes people use their positions in society not to queue up or follow procedure *Ba somebod*y making us to wait even more.

**I: How long have you been accessing health insurance?**

R: since last year 2023 officer from NHIMA followed us in the communities to register us the elderly and told us it’s free of charge no payments and I went with me with my wife’s NRC at the hospital so that they also register my wife under my account.

**I. Does your registration with the scheme cover all family members under your care?**

R: Only my wife as a beneficiary I was told she the only person I can add since am on a free account.

**I. Do you find the scheme affordable and helpful for the elderly citizens?**

R: Yes, very much of us we have worked for the country its now the country to take care of us.

**CHALLENGES OF NATIONAL HEALTH INSURANCE SCHEME**

**I: what challenges do you face when accessing health services from a NHIS facility?**

R: lack of medicine at hospital even when you are sent to a pharmacy still no medicine drugs is a huge challenge for NHIMA. Long queues are also another challenge waiting for long hours for one to be attended to.

**I: At the OPD 2, how will you rate the treatment you receive from the service providers NHIS officers?**

R: Officers take long since they must attend to a lot of people, others spend are slow and like using their phones. But sometimes the treatment is ok can get the service on time

**I: what challenges do face at the pharmacies when you take a prescription gotten from a NHIS hospital?**

R: we are forced to buy medicines if pharmacy says they also don’t have the medicine. Transport to pharmacy especially those that are far and sometimes when the pharmacy does not have you are forced to buy.

**I: Have you ever been told to pay for a medical service or drug that is not covered by the scheme? If yes how can this be avoided?**

R: NHIMA needs to work with pharmacies that are well stocked with medicines, NHIMA has a billing and claiming process these pharmacies need to give the medicines and claim the money from NHIMA

**I: What is your perception or observation about the NHIS in the Kitwe district?**

R: So far so good it is helping a lot it needs more support from the service providers and government

**I: What measures can government put in place to improve the scheme?**

R: Government needs to introduce a subsidy to help the vulnerable pay for their NHIMA scheme. Build more wards for NHIMA patients in government hospitals need to start working the high-cost section needs to have NHIMA people as well. Government needs to help NHIMA buy encouraging people to register on the health scheme, so everyone becomes a NHIMA member. Government needs to make sure that NHIMA is also introduced in clinics

**I: thank very much Sir we come to end of the interview**

**R: you are welcome**

**END OF INTERVIEW**

**Interview ID: 02**

**Interview Date: 6^th^ MAY 2024**

**Number of Participants: 01**

**Participant Title: NHIMA ELDERLY PRINCIPAL MEMBER (NURSE)**

**Site: Kitwe District**

**Transcriptionist: SAKALA LUCKY**

**DEMOGRAPHIC CHARACTERISTICS OF RESPONDENTS**

**I: Gender?**

R: Female

**I: How old are you Madam?**

R: I am a 58years old Zambia senior citizen

**I: What are you currently doing sir your occupation?**

R: Currently am working as a nurse working from Butondo clinic.

**I: Are you married madam?**

R: Yes, am a married woman

**I: how far have you going with your education sir?**

R: I went up to collage where I did my nursing

**ROLE OF THE NATIONAL HEALTH INSURANCE SCHEME IN STRENTHENING SERVICES DELIVELY AMONG THE ELDERLY**

**I: Do you access health care/ seek treatment from NHIMA hospital anytime you are sick without facing any difficulties?**

R: **Yes,** since I started no many challenges. Last time I was sick I was well attended to cause I went to private facility I was well taken care of reason being the private still have old health personal with experience as opposed to newly employed that are now found in government. I prefer private to government.

**I. How long have you been accessing health insurance?**

R: Since 2019 as we were registered by government when it was introduced since then they have been cutting from my pay

**I: Does your registration with the scheme cover all family members under your care?**

R: No just my husband and of my children, others are more than 18 and have their own personal NHIMA accounts.

**I: Do you find the scheme affordable and helpful for the elderly citizens?**

**R: No,** imagine some whose 60 and not doing anything they can’t pay the k30**.** They need to reduce the age to 55years for people that don’t make contributions Reduce the money to k15 more people will be paying than k30

**CHALLENGES OF NATIONAL HEALTH INSURANCE SCHEME**

**I: What challenges do you face when accessing health services from a NHIS facility?**

R: Lack of drugs, Pharmacies also have no medicines and forced to buy medicines

**I: At the OPD 2, how will you rate the treatment you receive from the service providers NHIS officers?**

R: Good especially if find a good officer you get good treatment other may have moods depending on the day

**I: What challenges do face at the pharmacies when you take a prescription gotten from a NHIS hospital?**

R: Since they deduct money drugs must be found at hospital pharmacy more drugs must be under NHIMA

**I: Have you ever been told to pay for a medical service or drug that is not covered by the scheme? If yes how can this be avoided?**

R: in my case I have never, mostly they cover everything Medicines must be available because we are being deducted every month.

**I: What is your perception or observation about the NHIS in the Kitwe district?**

R: Some facilities are slow due to the NHIMA system that mostly is down their area lot of procedures figure prints and people take a lot of time at the facility.

**I: what measures can government put in place to improve the scheme?**

R: government need to build ore facilities for NHIMA to accredit, we need more clinics under NHIMA. Government can make sure that hospitals under NHIMA stocks enough drugs and more manpower under NHIMA so that member do not bounce.

**I: thank very much madam we come to end of the interview**

R: you are welcome

**END OF INTERVIEW**

**Interview ID: 03**

**Interview Date: 6^th^ MAY 2024**

**Number of Participants: 01**

**Participant Title: NHIMA ELDERLY PRINCIPAL MEMBER (Farmer)**

**Interview Category: IDI NHC Kitwe Teaching Hospital**

**Site: Kitwe District**

**Transcriptionist: SAKALA LUCKY**

**DEMOGRAPHIC CHARACTERISTICS OF RESPONDENTS**

I: Gender**:**

R: Male

**I: How old are you Sir?**

R: I am 56years old

I: What are you currently doing Sir?

R: currently am working on my own am self employed

I: Are you married Sir

R: yes, I am married

I: How far have you gone with your education?

R: Secondary school level

**SECTION B.**

**ROLE OF THE NATIONAL HEALTH INSURANCE SCHEME IN STRENTHENING SERVICES DELIVELY AMONG THE ELDERLY**

**I: Do you access health care/ seek treatment from NHIMA hospital anytime you are sick without facing any difficulties? If the answer is No, what challenges do you face?**

R: NO When I travelled to Lusaka, they didn’t find the record in the system, they hard to call head office to check because the system was down that took a while but after confirming later, I was attended to.

**I: How long have you been accessing health insurance?**

R: I have been with NHIMA since 2019 just went it was introduced the company, I was working with registered me.

**I: Does your registration with the scheme cover all family members under your care?**

R: NO, my children more than 18years have their own account only my wife is under my Scheme

**I: Do you find the scheme affordable and helpful for the elderly citizens?**

R: No, they need to reduce the age to 55 for nonpaying citizens and not 65years, People who are working are very few now unless the times of ZCCM Elderly people need free medication reduce from 55 to 65 that will be affordable If someone retirees immediately they should access free services without any difficulties since people invest as they work. Through NHIMA every Zambian must access the services because a lot of people are working meaning it’s no longer government who’s buying medicines but people as well

**CHALLENGES OF NATIONAL HEALTH INSURANCE SCHEME**

**I: what challenges do you face when accessing health services from a NHIS facility?**

R: there several challenges for example crowds of people at the NHIMA section, long queues you must wait for a long time to be attended to. The System is very slow sometimes they take time looking for our records, the record keeping is very poor let those in public facilities learn how to keep records like private ones

**I: At the OPD 2, how will you rate the treatment you receive from the service providers NHIS officers?**

R: Very good I have seen enough manpower, and sometimes they are very fast if the system is down, they use paper claims.

**I: what challenges do face at the pharmacies when you take a priscription gotten from a NHIS hospital?**

R: the main challenge there is Lack of medicines and sometimes the attitude of the workers is very bad towards NHIMA clients.

**13. Have you ever been told to pay for a medical service or drug that is not covered by the scheme? If yes how can this be avoided**

R: No I have I usually go to other facilities if no medicine I buy from other pharmacies not NHIMA.

I: **What is your perception or observation about the NHIS in the Kitwe district?**

R: Out of 10 I can give it 7 am happy nhima is also private the attitude is very much ok happy stuff as compared to government facilities. Need to sensitize the officers in government no supervision and they feel their jobs are more secure, NHIMA needs a questionnaire to help the clients rates the work ethics of the facility

**I: what measures can government put in place to improve the scheme?**

R: Reduce the age from 65 to 55year for nonpaying clients Make sure medicines are available They need to assess and inspecting stuff attitudes toward NHIMA patients and all NHIMA facilities need serious inspection if the facility is environmentally friendly Encourage cleanness in the hospital especially in government facilities

**I: thank very much Sir we come to end of the interview**

R: you are welcome

**END OF INTERVIEW**

**Interview ID: 04**

**Interview Date: 7^th^ MAY 2024**

**Number of Participants: 01**

**Participant Title: NHIMA ELDERLY PRINCIPAL MEMBER (retiree)**

**Site: Kitwe District**

**Transcriptionist: SAKALA LUCKY**

DEMOGRAPHIC CHARACTERISTICS OF RESPONDENTS

**I: Gender**:

R: Male

**I: how old are you Sir**

R: I am 80years old

**I: what are you currently doing your occupation**

**R:** Currently am doing nothing am a retiree been helped by my children

I: what is your marital Status sir?

R: I am married

**I: what is your level of education**

R: secondary level

**SECTION B.**

**ROLE OF THE NATIONAL HEALTH INSURANCE SCHEME IN STRENTHENING SERVICES DELIVELY AMONG THE ELDERLY**

**I: Do you access health care/ seek treatment from nhima hospital anytime you are sick without facing any difficulties?**

R: NO, too many people causing more queues at the hospital. Too much time spent at the hospital and NHIMA stuff are a few

**I. How long have you been accessing health insurance?**

R: I joined in 2020 when people officers from NHIMA visited our church to register senior citizens

**I: Does your registration with the scheme cover all family members under your care?**

**R: Yes,** Me and my wife since I can’t add anyone else

**I: Do you find the scheme affordable and helpful for the elderly citizens?**

R: Yes, if possible, paying more is better to assist even those that can’t afford health is so expensive people need to invest and pay more for health services. Better you pay more by investing more when have an emergency no problem.

**CHALLENGES OF NATIONAL HEALTH INSURANCE SCHEME**

**I: what challenges do you face when accessing health services from a NHIS facility?**

R: No medicines at hospital and pharmacies NHIMA need to find a solution we walk to hospital stand in long queues only to be told no medicine you go to the pharmacy still nothing this is a challenge for us elderly. Other services like huge scans are only in Lusaka meaning I need transport to go that side this is to cost for me.

**I: At the OPD 2, how will you rate the treatment you receive from the service providers NHIS officers?**

R: Very good Officers are good but more manpower

**I: what challenges do face at the pharmacies when you take a prescription gotten from a NHIS hospital?**

R: I have BP in most cases the pharmacy will say no medicines here.

**I: Have you ever been told to pay for a medical service or drug that is not covered by the scheme**?

R: No but I have heard people sometimes are made to buy medicine if not found people will have no option but to get the medicine from their out-of-pocket money.

I: What is your perception or observation about the NHIS in the Kitwe district?

R: Distance to health facilities in the district especially for us elderly transport is a challenge

**I. what measures can government put in place to improve the scheme?**

R: Government must not involve politics in the scheme instead make for effective by funding the scheme more. More facilities under NHIMA need to be built.

**I: thank very much Sir we come to end of the interview**

R: you are welcome

**END OF INTERVIEW**

**Interview ID: 05**

**Interview Date: 7^th^ MAY 2024**

**Number of Participants: 1**

**Participant Title: NHIMA ELDERLY PRINCIPAL MEMBER (Farmer)**

**Site: Kitwe District**

**Transcriptionist: SAKALA LUCKY**

DEMOGRAPHIC CHARACTERISTICS OF RESPONDENTS

**I: Gender**:

R: Male

I: How old are you Sir?

R: I am 58years old

I: Occupation:

R: right now, am just working on my own self employed am working on my farm with my wife.

**I: What is your marital Status?**

R: I am married

**I: What is the level of your education?**

R: I reached college level

**ROLE OF THE NATIONAL HEALTH INSURANCE SCHEME IN STRENTHENING SERVICES DELIVELY AMONG THE ELDERLY**

**I: Do you access health care/ seek treatment from NHIMA hospital anytime you are sick without facing any difficulties?** □Yes □ No **If the answer is No what challenges do you face?**

R: NO NHIMA facilities are full of challenges number one lack of medicine each time you visit a pharmacy or hospital you find there no drugs at the facility. Number two long ques and long procedures one need to be patient with NHIMA which is bad for emergencies.

**I: How long have you been accessing health insurance?**

R: I joined in 2019 we were the first people to you NHIMA before it went to the public

**8. Does your registration with the scheme cover all family members under your care?**

R: Yes all my family members accept those more than 18years

**I: Do you find the scheme affordable and helpful for the elderly citizens?**

R: No looking at the economy and you doing nothing k30 is not affordable but if do something k30 is very much affordable in most cases elderly people are just sitting at home.

**CHALLENGES OF NATIONAL HEALTH INSURANCE SCHEME**

**I: what challenges do you face when accessing health services from a NHIS facility?**

R: NHIMA needs to go to clinics as well there near the people to cut on transport costs

Primary health care needs to near and under NHIMA.

**I: At the OPD 2, how will you rate the treatment you receive from the service providers NHIS officers?**

R: I can’t complain much they try to be nice and very good

**I: what challenges do face at the pharmacies when you take a prescription gotten from a NHIS hospital?**

R: Lack of medication most pharmacies stuff member looks immature and don’t have good attitude.

**I: Have you ever been told to pay for a medical service or drug that is not covered by the scheme?**

R: Yes, I was told to buy since they had no medicines, I had to buy the drug get well then, I visited the office in town to complain about my situation

**I: What is your perception or observation about the NHIS in the Kitwe district?**

R: They need to work on issuing of lenses for clients they take too long with bad flame

I: what measures can government put in place to improve the scheme?

NHIMA need to reach the clinic to cut on transport NHIMA needs pharmacies and drug stores and order their own medicines. Government needs to build more facilities that can be under NHIMA so that member can have a lot to choose from.

**I: thank very much Sir we come to end of the interview**

R: you are welcome

**END OF INTERVIEW**

**Interview ID: 06**

**Interview Date: 7^th^ MAY 2024**

**Number of Participants: 1**

**Participant Title: NHIMA ELDERLY PRINCIPAL MEMBER (Senior citizen)**

**Site: Kitwe District**

**Transcriptionist: SAKALA LUCKY**

**I: Gender R**: Female

**I: how old are you?**

R: I am a 68years old

**I: what is your Occupation madam?**

R: currently I am unemployed I depend on my children support

**I: what is your marital Status?**

R: I am widow

**I: What is the level of your education?**

R: primary level

**ROLE OF THE NATIONAL HEALTH INSURANCE SCHEME IN STRENTHENING SERVICES DELIVELY AMONG THE ELDERLY**

**I. Do you access health care/ seek treatment from NHIMA hospital anytime you are sick without facing any difficulties? If the answer is No, what challenges do you face?**

R: NO, there need separate NHIMA patients from the ordinary when you visit a government facility, for example central hospital NHIS clients are mixed with ordinary people all in one place.

**I. How long have you been accessing health insurance?**

R: I registered in when the near offices came going door to door in our compound in 2022

**I: Does your registration with the scheme cover all family members under your care?**

R: No am just alone since my spouse is late

**I: Do you find the scheme affordable and helpful for the elderly citizens?**

R: Yes, for me I don’t pay anything unless there no medicines that’s when I buy some, those that pay I hear k30 is the minimum which is ok if people can afford. NHIMA just needs improve its services especially government facilities

**CHALLENGES OF NATIONAL HEALTH INSURANCE SCHEME**

**I: What challenges do you face when accessing health services from a NHIS facility?**

R: At the facility we take long there a lot of people now under NHIMA so you can spend the whole day at the facility. We need a lot of facilities and a lot of people working under NHIMA

I**. At the OPD 2, how will you rate the treatment you receive from the service providers NHIS officers?**

R the treatment in most cases is good despite the time we spend waiting for one to get the services.

**I: What challenges do face at the pharmacies when you take a prescription gotten from a NHIS hospital?**

R: There was no medicine available at the pharmacy, me personally I have BP each time I go there no medicine as a result I buy so that I keep myself healthy.

**I: Have you ever been told to pay for a medical service or drug that is not covered by the scheme?**

R: yes like I said once there no medicines and you are sick better you buy the medicines yourself unless you want to die.

**I: What is your perception or observation about the NHIS in the Kitwe district?**

R: in Kitwe the services are improving we have good meals under NHIMA but compared to Ndola, I can say Ndola is much better there you have NHIMA wards you don’t mix with other people that’s how it must be and Kitwe needs that too.

**I. What measures can government put in place to improve the scheme?**

R: NHIMA is good thing that needs government support financially, there is need for more facilities especially clinics that are near the people offering primary health care. More health personals need to be employed by the government so that we have enough people to work under NHIMA in government hospitals.

**I: thank very much madam we come to end of the interview**

R: you are welcome

**END OF INTERVIEW**

**Interview ID: 07**

**Interview Date: 8^th^ MAY 2024**

**Number of Participants: 1**

**Participant Title: NHIMA ELDERLY PRINCIPAL MEMBER (Farmer)**

**Site: Kitwe District**

**Transcriptionist: SAKALA LUCKY**

**DEMOGRAPHIC CHARACTERISTICS OF RESPONDENTS**

**I: Gender:**

R: Female

**I: How old are you madam?**

R: I am 66 years old

**I: what are you currently doing your occupation**?

R: I am currently farming at a small scale.

I: What is your Marital Status?

R: Married

**I: What is the level of your education?**

R: secondary level

**SECTION B.**

**ROLE OF THE NATIONAL HEALTH INSURANCE SCHEME IN STRENTHENING SERVICES DELIVELY AMONG THE ELDERLY**

**I: Do you access health care/ seek treatment from NHIMA hospital anytime you are sick without facing any difficulties? □Yes □ No If the answer is No what challenges do you face?**

R: government facilities have challenges of long queues we take time when we visit the facilities, in most cases the diet is poor we are mixed with other patients NHIMA needs to improve its services and sometimes you are told there No medicines available.

**I. How long have you been accessing health insurance?**

R: we registered at the Catholic Church NHIMA officers visited us in 2022 for senior citizens registration

**I: Does your registration with the scheme cover all family members under your care?**

R: No am just alone on my scheme my 1st born and 2nd are also on NHIMA

**I. Do you find the scheme affordable and helpful for the elderly citizens?**

R: No people are struggling they need to reduce to 55years for one to stop paying. People pay minimum k30 it sounds low but the economy is very bad now so me I think people that are 55years old should not pay NHIMA.

**CHALLENGES OF NATIONAL HEALTH INSURANCE SCHEME**

**I. what challenges do you face when accessing health services from a NHIS facility?**

R: with NHIMA the process is too long you need to wait for approval from Lusaka if need a scan services if it’s lenses one has to wait for two weeks meanwhile you are in pain with your eyes. There just too many delays that need serious attention from the NHIMA part.

**I: At the OPD 2, how will you rate the treatment you receive from the service providers NHIS office?**

R: The treatment is good the officers really try their best to serve us but there a lot of clients that need services NHIMA need a lot of employees

**I: what challenges do face at the pharmacies when you take a prescription gotten from a NHIS hospital?**

R: Lack of medicines these facilities under NHIMA disappoint us a lot after using your transport money taking the prescription that side you just hear no medicine or NHIMA is not paying us so we have stopped giving medicines.

**I: Have you ever been told to pay for a medical service or drug that is not covered by the scheme?**

R: Yes I buy me BP medicines got tired of the same answers no medicine, NHIMA not paying us

I: What is your perception or observation about the NHIS in the Kitwe district?

If I compare it to Mansa Kitwe is better, in Mansa there no facilities just the hospital which is also flooded with people NHIMA needs to better its services.

I: What measures can government put in place to improve the scheme?

Good needs to support NHIMA financially to make medicines available to its clients people are suffering some that don’t have money to buy may even die. Government needs to make sure NHIMA reduces on the long process it has it needs to be approving fast so that people get the services.

**I: thank very much madam we come to end of the interview**

R: you are welcome

**END OF INTERVIEW**

**Interview ID: 08**

**Interview Date: 8^th^ MAY 2024**

**Number of Participants: 1**

**Participant Title: NHIMA ELDERLY PRINCIPAL MEMBER (Farmer)**

**Site: Kitwe District**

**Transcriptionist: SAKALA LUCKY**

DEMOGRAPHIC CHARACTERISTICS OF RESPONDENTS

**I. Gender:**

Male

**I: How old are you Sir**?

R: I am 64 years

**I: What are u currently doing?**

R: I work on my own self employed, working on my small farm

I: what is your marital status?

R: I am married

I what is your level of education?

R: secondary level

**SECTION B.**

**ROLE OF THE NATIONAL HEALTH INSURANCE SCHEME IN STRENTHENING SERVICES DELIVELY AMONG THE ELDERLY**

**I: Do you access health care/ seek treatment from nhima hospital anytime you are sick without facing any difficulties? □Yes □ No. If the answer is No what challenges do you face**

R: No personally I feel NHIMA needs to do more we need wards not mixing us with non-members, the lack of drugs at the hospital is a huge challenge even when you sent to a pharmacy you get the same response no medicines.

**I: How long have you been accessing health insurance?**

R: In 2022 I was registered by NHIMA officers going door to door in our compound and I was told to pay k30 until next year when I reach 65years.

**I: Does your registration with the scheme cover all family members under your care?**

R: No just me and my wife my children are registered from their work places

**I: Do you find the scheme affordable and helpful for the elderly citizens?**

R: No a lot of elderly people are not doing anything imagine that k30 you don’t have or you pay but find no medicines at the hospital, once one is 60years its better they stop paying.

**CHALLENGES OF NATIONAL HEALTH INSURANCE SCHEME**

I: What challenges do you face when accessing health services from a NHIS facility?

R: at the hospital we are told wait for NHIMA to approve this takes long than expected imagine you have an emergency that needs immediate attention. And the lack of medicines.

**I: At the OPD 2, how will you rate the treatment you receive from the service providers NHIS officers?**

R: Good no much problem from them just add more stuff NHIMA has huge numbers so adding more people will help us because when you go to the hospital you can spend the whole day because of a lot of numbers and a few workers to serve the people.

**I: what challenges do face at the pharmacies when you take a prescription gotten from a NHIS hospital?**

R: For me sometimes I am able to collect medicine without challenges which I appreciate NHIMA for.

**I. Have you ever been told to pay for a medical service or drug that is not covered by the scheme?**

R: yes but I didn’t buy I just walked away I had some extra at home

**I: What is your perception or observation about the NHIS in the Kitwe district?**

R: Private hospitals are better than government under NHIMA when it comes to treatment that’s why we need a lot of good private facilities under NHIS

**I: What measures can government put in place to improve the scheme?**

R: we need more nhima wards and avoid mixing members with non-members, secondly, more NHIMA stuff is needed we now a lot we need to be served by more people not a few and more medication from government to avoid shortages

**I: thank very much Sir we come to end of the interview**

R: you are welcome

**END OF INTERVIEW**

**Interview ID: 09**

**Interview Date: 9^th^ MAY 2024**

**Number of Participants: 1**

**Participant Title: NHIMA ELDERLY PRINCIPAL MEMBER (Senior Citizen)**

**Site: Kitwe District**

**Transcriptionist: SAKALA LUCKY**

**DEMOGRAPHIC CHARACTERISTICS OF RESPONDENTS**

**I: Gender**

R; Female

**I: what is your age range?**

R: I am 69years old

**I: what are you currently doing your Occupation?**

R: currently I just sit at home unemployed

**I: what is your Marital Status?**

R; I am Married my husband is gone at the farm

**I: what is your level of education?**

R: I reached primary level

SECTION B.

**ROLE OF THE NATIONAL HEALTH INSURANCE SCHEME IN STRENTHENING SERVICES DELIVELY AMONG THE ELDERLY**

**I: Do you access health care/ seek treatment from nhima hospital anytime you are sick without facing any difficulties? If the answer is No what challenges do you face?**

R: No I experienced long ques there a lot of people under NHIMA now so we take long at the hospital and the stuff under NHIMA is under stuffed we need more people working there.

**I. How long have you been accessing health insurance?**

R: last year 2023 that is when I joined and became a member of the scheme we registered from our church

**I: Does your registration with the scheme cover all family members under your care?**

R: No am alone my husband also has his own account

**I: Do you find the scheme affordable and helpful for the elderly citizens?**

R: NO for me it’s free but my friends who are 60years they pay 30k which not fair to them they are doing nothing just home elderly people need free health services

**CHALLENGES OF NATIONAL HEALTH INSURANCE SCHEME**

**I: what challenges do you face when accessing health services from a NHIS facility?**

R: lack of medicine at hospital even when you are sent to a pharmacy still no medicine drugs is a huge challenge for NHIMA. Long queues are also another challenge waiting for long hours for one to be attended to.

**I: At the OPD 2, how will you rate the treatment you receive from the service providers NHIS officers?**

R: Officers take long since they must attend to a lot of people, Others spend are slow and like using their phones. But sometimes the treatment is ok can get the service on time

**I: what challenges do face at the pharmacies when you take a priscription gotten from a NHIS hospital?**

R: we are forced to buy medicines if pharmacy says they also don’t have the medicine. Transport to pharmacy especially those that are far and sometimes when the pharmacy does not have you are forced to buy.

**I: Have you ever been told to pay for a medical service or drug that is not covered by the scheme? If yes how can this be avoided?**

R: NHIMA needs to work with pharmacies that are well stocked with medicines, NHIMA has a billing and claiming process these pharmacies need to give the medicines and claim the money from nhima

I: What is your perception or observation about the NHIS in the Kitwe district?

R: So far so good it is helping a lot it needs more support from the service providers and government its better compared to kalulushi.

**I: What measures can government put in place to improve the scheme?**

R: Government needs to introduce a subsidy to help the vulnerable pay for their NHIMA scheme. Build more wards for NHIMA patients in government hospitals need to start working the high-cost section needs to have NHIMA people as well. Government needs to help NHIMA buy encouraging people to register on the health scheme, so everyone becomes a NHIMA member. Government needs to make sure that nhima is also introduced in clinics near people.

**I: thank very much madam we come to end of the interview**

R: you are welcome

**END OF INTERVIEW**

**Interview ID: 10**

**Interview Date: 10^th^ MAY 2024**

**Number of Participants: 1**

**Participant Title: NHIMA ELDERLY PRINCIPAL MEMBER (retiree)**

**Site: Kitwe District**

**Transcriptionist: SAKALA LUCKY**

**DEMOGRAPHIC CHARACTERISTICS OF RESPONDENTS**

**I: Gender?**

R: Female

**I: How old are you Madam?**

R: I am a 70years old Zambia senior citizen

**I: What are you currently doing sir your occupation?**

R: Retired government worker

**I: Are you married madam?**

R: Am a widow

**I: how far have you going with your education sir?**

R: I reached secondary level

**SECTION B.**

**ROLE OF THE NATIONAL HEALTH INSURANCE SCHEME IN STRENTHENING SERVICES DELIVELY AMONG THE ELDERLY**

I: Do you access health care/ seek treatment from nhima hospital anytime you are sick without facing any difficulties? □Yes □ No

**R: Yes** for now I can say they are helping

**I: How long have you been accessing health insurance?**

Since 2023 when we joined from our church the officers visited us.

**I: Does your registration with the scheme cover all family members under your care?**

No am just alone others need to pay so they have no nhima accounts

**I: Do you find the scheme affordable and helpful for the elderly citizens?**

R: Yes it’s very important to fund the scheme so that us we benefit us the elderly ones.

**CHALLENGES OF NATIONAL HEALTH INSURANCE SCHEME**

**I: what challenges do you face when accessing health services from a NHIS facility?**

R: we spend long hours at the hospitals due to few numbers of the members of stuff and the lack of medicines at the facilities.

**I: At the OPD 2, how will you rate the treatment you receive from the service providers NHIS officers?**

Very good we elderly sometimes we are given priority which good treatment from the stuff officers, key is to I follow instructions.

**I: what challenges do face at the pharmacies when you take a prescription gotten from a NHIS hospital?**

R: Lack of medicines at the pharmacy they also don’t have the medicine. Transport to pharmacy especially those that are far and sometimes when the pharmacy does not have you are forced to buy.

**I: Have you ever been told to pay for a medical service or drug that is not covered by the scheme?**

R: Yes I buy me BP medicines got tired of the same answers no medicine, NHIMA not paying us

I: What is your perception or observation about the NHIS in the Kitwe district?

If I compare it to Mansa Kitwe is better, in Mansa there no facilities just the hospital which is also flooded with people NHIMA needs to better its services.

I: What measures can government put in place to improve the scheme?

Good needs to support NHIMA financially to make medicines available to its clients people are suffering some that don’t have money to buy may even die. Government needs to make sure NHIMA reduces on the long process it has it needs to be approving fast so that people get the services.

**I: thank very much madam we come to end of the interview**

R: you are welcome

**END OF INTERVIEW**

**Interview ID: 11**

**Interview Date: 25^th^ MAY 2024**

**Number of Participants: 1**

**Participant Title: NHIMA ELDERLY PRINCIPAL MEMBER (farmers)**

**Site: Kitwe District**

**Transcriptionist: SAKALA LUCKY**

**DEMOGRAPHIC CHARACTERISTICS OF RESPONDENTS**

I: Gender?

R: Male

**I: How old are you sir?**

R: I am 69years old

**I: what are you currently doing?**

R: Right now, am self-employed after working for the mines for 26years. Farming is what is helping me now

**I: What is my current Marital Status?**

R: I am married

**I: what the level of your education Sir?**

R: form 5 secondary level

**SECTION B.**

**ROLE OF THE NATIONAL HEALTH INSURANCE SCHEME IN STRENTHENING SERVICES DELIVELY AMONG THE ELDERLY**

**I: Do you access health care/ seek treatment from nhima hospital anytime you are sick without facing any difficulties? □Yes □ No If the answer is No what challenges do you face?**

R: No mostly there long ques which is not good for us elderly ones as much priority is give in most cases we are told to wait for hours before we get the service.

I: How long have you been accessing health insurance?

R: Since 2021 when NHIMA just started they registered us the mines.

**I: Does your registration with the scheme cover all family members under your care?**

R: Since am a senior citizen only my wife is on my account

I: Do you find the scheme affordable and helpful for the elderly citizens?

R: yes personally I don’t pay but those that pay even k30 is ok one can try to organize a k30 remember health is very expensive.

**CHALLENGES OF NATIONAL HEALTH INSURANCE SCHEME**

**I: what challenges do you face when accessing health services from a NHIS facility?**

R: No medication even at the pharmacies this one is huge challenge for us especially that most times we are broke.

**I: At the OPD 2, how will you rate the treatment you receive from the service providers NHIS officers?**

R: The treatment is every good especially with private facilities, the only problem is the stuff are a few and NHIMA now has huge numbers of people.

**I: what challenges do face at the pharmacies when you take a prescription gotten from a NHIS hospital?**

R: Lack of medicines and bad attitude

**I: Have you ever been told to pay for a medical service or drug that is not covered by the scheme?**

R: No me I usually get the medicines but I have heard people complaining of not getting.

**I: What is your perception or observation about the NHIS in the Kitwe district?**

R: There a lot of complaints NHIMA needs to work on that needs attention especially bringing on board good facilities though Kitwe is better than Chingola and other districts

**I what measures can government put in place to improve the scheme?**

R: we need more nhima wards and avoid mixing members with non-members, secondly, more NHIMA stuff is needed we now a lot we need to be served by more people not a few and more medication from government to avoid shortages

**I: thank very much Sir we come to end of the interview**

R: you are welcome

**END OF INTERVIEW**

**Interview ID: 12**

**Interview Date: 25^th^ MAY 2024**

**Number of Participants: 1**

**Participant Title: NHIMA ELDERLY PRINCIPAL MEMBER (Farmer)**

**Site: Kitwe District**

**Transcriptionist: SAKALA LUCKY**

**DEMOGRAPHIC CHARACTERISTICS OF RESPONDENTS**

**I: Gender**

R: Male

**I: What is your age range?**

R: I am 77years old

**I: what are you currently doing?**

I am self-employed after the mines I start doing farming just for consumption

**I: What is your Marital Status?**

R: am married my wife has gone to the market.

**I: What is your level of education?**

R: I reached form V Secondary level

**SECTION B.**

**ROLE OF THE NATIONAL HEALTH INSURANCE SCHEME IN STRENTHENING SERVICES DELIVELY AMONG THE ELDERLY**

**I: Do you access health care/ seek treatment from nhima hospital anytime you are sick without facing any difficulties? □Yes □ No If the answer is No what challenges do you face?**

**R: No it** takes long to see a doctor and they’re very few doctors under NHIMA making it a problem for people to have services on time. Lack of medication is another challenge at the facilities.

**I: How long have you been accessing health insurance?**

R: Since 2021 when NHIMA started we were registered by the company

**I: Does your registration with the scheme cover all family members under your care?**

**R:** yes me and my spouse

I: Do you find the scheme affordable and helpful for the elderly citizens?

Yes k30 is a good amount people can run up and down find the K30 and pay some of us are over 65 so we can’t

**CHALLENGES OF NATIONAL HEALTH INSURANCE SCHEME**

**I: what challenges do you face when accessing health services from a NHIS facility?**

Lack of medicines and Long queues for us to wait to be attended to.

**I: At the OPD 2, how will you rate the treatment you receive from the service providers NHIS officers?**

The officers try a lot but they are very few so the treatment is very good.

**I: What challenges do face at the pharmacies when you take a prescription gotten from a NHIS hospital?**

Lack of medicine and system failure NHIMA system is problem sometimes you can have all the requirements but the system can fail us sometimes which is a huge challenge.

**I: Have you ever been told to pay for a medical service or drug that is not covered by the scheme?**

Yes there was a time I had my blood pressure high we found no medicines I had to buy which was so inconveniencing on my part

**I: What is your perception or observation about the NHIS in the Kitwe district?**

Kitwe needs to improve NHIMA services it has facilities but I feel we need more and more facilities to help out.

**I: What measures can government put in place to improve the scheme?**

R: Government has a role to play this health NHIMA needs funding and needs more health personal in order to serve its clients. NHIMA needs to make sure all its facilities have enough medicines so that we don’t bounce when we visit the facilities.

**I: thank very much Sir we come to end of the interview**

R: you are welcome

**END OF INTERVIEW**

**Interview ID: 13**

**Interview Date: 26^th^ MAY 2024**

**Number of Participants: 1**

**Participant Title: NHIMA ELDERLY PRINCIPAL MEMBER**

**Site: Kitwe District**

**Transcriptionist: SAKALA LUCKY**

**SECTION A.**

**DEMOGRAPHIC CHARACTERISTICS OF RESPONDENTS**

**I: Gender**

R: Female

**I: How old are you?**

R: I am 73years old

**I: what are you currently doing your occupation?**

R: Currently am unemployed I depend on my working children for survival.

**I: what is your current marital Status?**

R: My husband is late am a widow

**I: What is your level of education?**

R: Secondary level

**SECTION B.**

**ROLE OF THE NATIONAL HEALTH INSURANCE SCHEME IN STRENTHENING SERVICES DELIVELY AMONG THE ELDERLY**

**I: Do you access health care/ seek treatment from NHIMA hospital anytime you are sick without facing any difficulties? □Yes □ No If the answer is No what challenges do you face?**

R: No, long queues which is a huge problem for us the elderly each time you go to a NHIMA hospital you have to wait before someone attends to you. Transport to the pharmacy if the hospital has no medicine is another challenge in most cases us we do not have enough money.

I: **How long have you been accessing health insurance?**

R: Since 2023 last year we have a gathering at church where we were registered as members of NHIMA

**I: Does your registration with the scheme cover all family members under your care?**

R: Yes, me and my spouse

**I: Do you find the scheme affordable and helpful for the elderly citizens?**

R: Yes, personally I don’t make payments but k30 payment which is the minimum is ok health is expensive paying k30 every month is affordable especially for the elderly.

**CHALLENGES OF NATIONAL HEALTH INSURANCE SCHEME**

**I: What challenges do you face when accessing health services from a NHIS facility?**

R: Lack of medication and few members of stuff under NHIMA, even the number of facilities under NHIMA are very few looking at the number of people under NHIMA more needs to be done.

**I: At the OPD 2, how will you rate the treatment you receive from the service providers NHIS officers?**

R: Very good the few stuff members try by all means to help and serve us on time it is really a huge task on them numbers under NHIMA have really gone up.

**I: What challenges do face at the pharmacies when you take a prescription gotten from a NHIS hospital?**

R: Lack of medicines each time I visit the facility, bad attitude from the stuff members others even say NHIMA is not paying us.

**I: Have you ever been told to pay for a medical service or drug that is not covered by the scheme?**

R: No once am told no medicines I leave and try out the other facility if it is out of stock am forced to buy from somewhere not under NHIMA.

**I: What is your perception or observation about the NHIS in the Kitwe district?**

R: Improve more services it’s a very good scheme that needs government support and ensuring its delivering to the citizens.

**I: What measures can government put in place to improve the scheme?**

R: More medicines new facilities and more wards government need to invest more in NHIMA make sure it has enough facilities to help people, a lot of NHIMA personnel to serve the increasing membership that NHIMA keeps on registering every day.

**I: thank very much madam we come to end of the interview**

R: you are welcome

**END OF INTERVIEW**

**Interview ID: 14**

**Interview Date:28^th^ MAY 2024**

**Number of Participants: 01**

**Participant Title: NHIMA ELDERLY PRINCIPAL MEMBER (marketeer)**

**Site: Kitwe District**

**Transcriptionist: SAKALA LUCKY**

**DEMOGRAPHIC CHARACTERISTICS OF RESPONDENTS**

**I: Gender?**

R: Female

**I: How old are you Madam?**

R: I am a 72years old Zambia senior citizen

**I: What are you currently doing sir your occupation?**

R: currently I just do small scale farming and sale tomatoes

**I: Are you married madam?**

R: Am a widow

**I: how far have you going with your education sir?**

R: I reached secondary level

**SECTION B.**

**ROLE OF THE NATIONAL HEALTH INSURANCE SCHEME IN STRENTHENING SERVICES DELIVELY AMONG THE ELDERLY**

I: Do you access health care/ seek treatment from nhima hospital anytime you are sick without facing any difficulties? □Yes □ No

**R: Yes** for now I can say they are helping

**I: How long have you been accessing health insurance?**

Since 2023 when we joined from our church the officers visited us.

**I: Does your registration with the scheme cover all family members under your care?**

No am just alone others need to pay so they have no nhima accounts

**I: Do you find the scheme affordable and helpful for the elderly citizens?**

R: Yes it’s very important to fund the scheme so that us we benefit us the elderly ones.

**CHALLENGES OF NATIONAL HEALTH INSURANCE SCHEME**

**I: what challenges do you face when accessing health services from a NHIS facility?**

R: we spend long hours at the hospitals due to few numbers of the members of stuff and the lack of medicines at the facilities.

**I: At the OPD 2, how will you rate the treatment you receive from the service providers NHIS officers?**

Very good we elderly sometimes we are given priority which good treatment from the stuff officers, key is to I follow instructions.

**I: what challenges do face at the pharmacies when you take a prescription gotten from a NHIS hospital?**

R: Lack of medicines at the pharmacy they also don’t have the medicine. Transport to pharmacy especially those that are far and sometimes when the pharmacy does not have you are forced to buy.

**I: Have you ever been told to pay for a medical service or drug that is not covered by the scheme?**

R: Yes I buy me BP medicines got tired of the same answers no medicine, NHIMA not paying us

I: What is your perception or observation about the NHIS in the Kitwe district?

If I compare it to Luangwa Kitwe is better, in Mansa there no facilities just the hospital which is also flooded with people NHIMA needs to better its services.

I: What measures can government put in place to improve the scheme?

Good needs to support NHIMA financially to make medicines available to its clients people are suffering some that don’t have money to buy may even die. Government needs to make sure NHIMA reduces on the long process it has it needs to be approving fast so that people get the services.

**I: thank very much madam we come to end of the interview**

R: you are welcome

**END OF INTERVIEW**

**INTERVIEW GUIDE FOR SERVICE PROVIDERS OF NATIONAL HEALTH INSURANCE SCHEME AT NHIMA FACILITIES IN KITWE DISTRICT**

**Interview ID: 15**

**Interview Date: 3^rd^ JUNE 2024**

**Number of Participants: 01**

**Participant Title: In-charge supervisor Under NHIMA**

**Site: Kitwe Progress Medical centre ( Kitwe District)**

**Transcriptionist: SAKALA LUCKY**

**I: As a facility for how long have you been offering NHIMA services and how has been your experience so far?**

R: We have been offering NHIMA services since December of 2019 as progress medical center our experience has been good we have enjoyed working with NHIMA despite a few challenges here and there.

**I: approximately how many elderly NHIS clients do you attend to in a day?**

R: our facility sees several clients as for the elderly I can say we attended to 15 to 20 elderly people on a busy day that is elderly clients only though for everyone we attend to a lot of people both old and young.

**I: When it comes to long queues** **how you do help the elderly so that they don’t stay too long in the queues?**

R: Since there are elderly and mostly senior citizens, priority is given to them by prioritizing them so that they are attended to earlier look at their age.

I: **how do you help elderly clients in case the facility has no medication or certain scan service that is needed by an elderly client?**

**R:** A referral form is given to them to go to the nearest accredited facility where they find the medication, in case they complain about distance or in case they don’t find the medicines we advise they buy using their out-of-pocket money.

**I: What challenges do you face with elderly clients in providing services to them? Please explain?**

R: With the elderly sometimes it’s difficult to prioritize them in case you have an emergency case. For example, a maternity case and like it becomes difficult to attend to them first and not the pregnant woman. Sometimes it’s difficult to explain to them a procedure they need to follow using local language especially those that don’t understand bemba which is like the language used here. Some come to complain when they don’t find the medicines available at the pharmacy. Some facilities do not have or stock medicines, for example cancer medicines unless the cancer disease hospital in Lusaka.

**I: Once you offer services to NHIMA client do face challenges claiming your money from NHIMA?**

R: Sometimes NHIMA takes long to pay claims especially Manuel claim as compared to online. For example, if we use manual claims they take long as compared when members login and use their figure prints for billing when the system is up.

**I: If yes kindly explain the experience?**

**R:** For example if we use manual claims they take long as compared when members login and use their figure prints for billing when the system is up. Paper claim make take a week or two longer than billing online

**I: Looking at number of elderly people that do not contribute to the scheme how can government help NHIMA provide quality services to the nonpaying elderly members?**

R: Government needs to provide a fund specifically for nonpaying members that can help with their medical bills.

Government can partner with organizations providing health care to provide health services to the elderly for example Dr Agrawal’s who offer free optician services to elderly members.

Government can also provide a subsidy which especially for the health of NHIMA elderly members even those on social cash transfer.

**I: What effective measure can the government put in place to make sure the NHIS is sustainable despite having both contributing and noncontributing members?**

R: Ensure that NHIMA accredit more facilities, employees more health personal to help in NHIMA public facilities, monitor and make sure NHIMA is delivery quality services, ensure that NHIMA services reach the rural areas by building more facilities and accrediting to NHIMA.

**I: thank very much Sir we come to end of the interview**

R: you are welcome

**END OF INTERVIEW**

**Interview ID: 16**

**Interview Date: 3^rd^ JUNE 2024**

**Number of Participants: 01**

**Participant Title: Member Service Assistant**

**Site: Site: Kitwe Progress Medical Center ( Kitwe District)**

**Transcriptionist: SAKALA LUCKY**

**I: As a facility for how long have you been offering NHIMA services and how has been your experience so far?**

R: since December of 2019 as progress medical center our experience has been good, we have enjoyed working with NHIMA.

**I: Approximately how many elderly NHIS clients do you attend to in a day?**

R: During a busy day even 26 on slow one 10 to 15 elderly as you know our facility is very busy everyone wants to go to progress because its private.

**I: When it comes to long ques how do you help the elderly so that they don’t stay too long in the queues?**

R: In most cases priority is given to them looking at the age we usually ask the other clients to squeeze but if we have an emergency, we ask them to wait for some time.

**I: How do you help elderly clients in case the facility has no medication or certain scan service that is needed by an elderly client?**

R: Firstly, not all medicines are on NHIMA package some are not there, one needs to read and understand the package. The other thing is some medicines we don’t stock them but other pharmacies who not on NHIMA have so we ask to please buy if there in need otherwise its beyond us

**I: What challenges do you face with elderly clients in providing services to them? Please explain?**

Since they are elderly, some don’t like to wait, they complain even when the situation is beyond us. Most of them have duplicates in the system so we tell them to visit NHIMA office they complain again plus mostly sometimes the medicines they want may not be available.

**I: Once you offer services to NHIMA client do face challenges claiming your money from NHIMA? Yes, If yes kindly explain the experience?**

As a private facility when we use manual claims NHIMA takes time to pay us more reason we love to bill online. My experience with paper claims money delays to come and when they sent late NHIMA takes time to work on them so that they pay you on time meanwhile you are attending to their clients.

**I: Looking at the number of elderly people that do not contribute to the scheme how can the government help NHIMA provide quality services to the nonpaying elderly members?**

R: Firstly, the government needs to give NHIMA a fund that can help with members that do not contribute. Secondly government can encourage NHIMA to work close with stakeholders that offer health services to cater for the elderly citizens and Government through social cash transfer can also be contributing some funds to NHIMA as part of health sustainability

**I: what effective measure can government put in place to make sure the NHIS is sustainable despite having both contributing and noncontributing members?**

Build more NHIMA accredit more facilities in rural areas

Employees more health personal to help in NHIMA public facilities

Monitor and make sure NHIMA is delivery quality services looking at monies they are getting from the contributing members.

**I: thank very much, we come to end of the interview**

R: you are welcome

**END OF INTERVIEW**

**Interview ID: 17**

**Interview Date: 4^th^ MAY 2024**

**Number of Participants: 01**

**Participant Title: Customer CARE CLEAK**

**Site: Kitwe ROYAL Medical Centre Kitwe District**

**Transcriptionist: SAKALA LUCKY**

**I: As a facility for how long have you been offering NHIMA services and how has been your experience so far?**

We joined NHIMA in May 2022 so far, I can say our experience has been good.

**I: Approximately how many elderly NHIS clients do you attend to in a day?**

On a busy day we usually attend to 15 but mostly 10 or less the elderly ones

**I: When it comes to long ques how do you help the elderly so that they don’t stay too long in the queues?**

Elderly people are senior citizens of the country and need to give them priority when it comes to giving them services, we try to attend to them before others so that they can quickly finish and go home.

**I: how do you help elderly clients in case the facility has no medication or certain scan services that is needed by an elderly client**?

R: In most cases we try to tell them to visit another NHIMA facility nearby if we don’t have the medicines, sometimes we try to give them a drug that works in a similar way but mostly we tell to try other facilities or if they have money to buy the drug from other facilities that have.

**I: What challenges do you face with elderly clients in providing services to them?**

For us the elderly patients can be problematic sometimes for example they may want to be first even when there is an emergency to attend to. Some fail to understand if say no medication they say you are eating NHIMA funds. We also experience duplicates incorrect date of birth, so we ask them to visit NHIMA office which is a problem to them

I: **Once you offer services to NHIMA client do face challenges claiming your money from NHIMA? yes** If yes kindly explain the experience?

R: Sometimes if there are no system, we use Manuel claim, but these have proven to be delayed when it comes to payment this was common last year but for now we are ok.

**I: Looking at number of elderly people that do not contribute to the scheme how can government help NHIMA provide quality services to the nonpaying elderly members?**

The government needs to fund NHIMA more so that it pays for them services we give elderly members. NHIMA can partner with other organization that offer services to the elderly

**I: what effective measure can government put in place to make sure the NHIS is sustainable despite having both contributing and noncontributing members?**

We need NHIMA facilities here in Kitwe and other places if people are paying let them build and employee a lot of NHIMA stuff. Government needs to monitor the services that NHIMA is offering so that there is no compromise, but they need to fund it too.

**I: thank very much, we come to end of the interview**

R: you are welcome

**END OF INTERVIEW**

**Interview ID: 18**

**Interview Date: 4^th^ JUNE 2024**

**Number of Participants: 01**

**Participant Title: IN-CHARGE NHIMA SECTION**

**Site: Wusakile Mine Hospital (Kitwe District)**

**Transcriptionist: SAKALA LUCKY**

**I: As a facility for how long have you been offering NHIMA services and how has been your experience so far?**

Since last year February 2023 this is our second year of serving NHIMA clients.

**I: Approximately how many elderly NHIS clients do you attend to in a day?**

Our hospital is very busy but for elderly I say 25 on busy day maybe 15 on a slow day

**I: When it comes to long ques how do you help the elderly so that they don’t stay too long in the ques?**

been a busy facility especially NHIMA section we try to put them first but, in most cases, they wait for some time

**I: how do you help elderly clients in case the facility has no medication or certain scan service that is needed by an elderly client?**

We send them to the nearest facility under NHIMA or ask them to buy if they have money on them if not, they go to NHIMA pharmacy

**I: What challenges do you face with elderly clients in providing services to them? Please explain?**

They complain of too many people long ques, they doctors are slow and if you tell them no medication they will complain about transport to the nearest pharmacy

**I: Once you offer services to NHIMA client do face challenges claiming your money from NHIMA?**

R: NO, Mostly NHIMA pays us on time maybe it’s because we under government if they delay it’s a day or two

**I: Looking at number of elderly people that do not contribute to the scheme how can government help NHIMA provide quality services to the nonpaying elderly members?**

R: NHIMA needs a lot of funding. Health care is expensive, so government needs to help with monies and NHIMA needs to partner with other organizations to ensure that it gives the elderly proper health services.

**I: what effective measure can government put in place to make sure the NHIS is sustainable despite having both contributing and noncontributing members?**

R: Employees more health personal to work in NHIMA hospitals especially in rural areas.

Build more facilities and make sure that NHIMA services are found in most parts of the country not just Copper belt and Lusaka old people are in villages they need good health as well

**I: thank very much, we come to end of the interview**

R: you are welcome

**END OF INTERVIEW**

**Interview ID: 19**

**Interview Date: 4^th^ JUNE 2024**

**Number of Participants: 01**

**Participant Title: CUSTOMER SERVICES OFFICER**

**Site: Kano Dental medical Center (Kitwe District)**

**Transcriptionist: SAKALA LUCKY**

**I: As a facility for how long have you been offering NHIMA services and how has been your experience so far?**

We been offering NHIMA services since 2022 November

**I: Approximately how many elderly NHIS clients do you attend to in a day?**

R: Our facility is small we have 10 to 15 on a busy day maybe 5 on a slow day

**When it comes to long ques how do you help the elderly so that they don’t stay too long in the ques?**

R: The elderly here are treated with priority. If we don’t have an emergency with the other members we try to attend to them as quickly as possible.

**I: how do you help elderly clients in case the facility has no medication or certain scan service that is needed by an elderly client?**

R: In most times when our elderly clients don’t find the drugs available, we ask them to visit the nearest NHIMA facility or if they have money, we ask them to buy from the nearest facility because the NHIMA pharmacy maybe far for them.

**I: What challenges do you face with elderly clients in providing services to them?**

R: Mostly the elderly need extra attention you need to listen carefully and need to explain to them the instructions repeated for them not to make a mistake. Sometimes they complain a lot once told the medication is not available. Some will ask you for transport once you tell them the medication can be found in the pharmacy in town.

**I: Once you offer services to NHIMA client do face challenges claiming your money from NHIMA?**

R: NO**,** NHIMA paying us though sometimes they delay some pharmacies belonging to my friends have stopped offering NHIMA services saying NHIMA takes long but in my case, they may delay but they pay us

**I: Looking at the number of elderly people that do not contribute to the scheme how can the government help NHIMA provide quality services to the nonpaying elderly members?**

R: Funding is key free is not free someone must be paying the government can increase the funding to NHIMA looking at how NHIMA is helping the elderly. Government can also help NHIMA by fostering partnerships between NHIMA and other health organization to help fund the senior citizen package.

**I: what effective measure can government put in place to make sure the NHIS is sustainable despite having both contributing and noncontributing members?**

R: We need more NHIMA facilities. We need NHIMA in clinics those are near the people like that old people will be walking to access healthcare. Government can monitor NHIMA operations making sure the monies are used for the intended purpose.

**I: thank very much, we come to end of the interview**

R: you are welcome

**END OF INTERVIEW**

**Interview ID: 20**

**Interview Date: 4^th^ JUNE 2024**

**Number of Participants: 01**

**Participant Title: ADMINISTRAVE ASSITANT**

**Site: kano dental medical Center (Kitwe District)**

**Transcriptionist: SAKALA LUCKY**

**I: As a facility for how long have you been offering NHIMA services and how has been your experience so far?**

R: We have been offering NHIMA services since 2022.

**I: approximately how many elderly NHIS clients do you attend in a day?**

R: On a busy day we usually attend to 15 but mostly 10 or less the elderly ones

**I: When it comes to long ques how do you help the elderly so that they don’t stay too long in the ques?**

R: Elderly people are senior citizens of the country and need to give them priority when it comes to giving them services, we try to attend to them before others so that they can quickly finish and go home.

**I: how do you help elderly clients in case the facility has no medication or certain scan services that are needed by an elderly client**?

R: In most cases we try to tell them to visit another NHIMA facility nearby if we don’t have the medicines, sometimes we try to give them a drug that works in a similar way but mostly we tell to try other facilities or if they have money to buy the drug from other facilities that have.

**I: What challenges do you face with elderly clients in providing services to them?**

R: For us elderly patients can be problematic sometimes, for example they may want to be first even when there is an emergency to attend to.

Some fail to understand if say no medication they say you are eating NHIMA funds.

We also experience duplicates incorrect date of birth, so we ask them to visit NHIMA office which is a problem to them.

**I: Once you offer services to NHIMA client do face challenges claiming your money from NHIMA? yes**

R: If yes kindly explain the experience? Sometimes if they are no system, we use Manuel claim, but these have proven to be delayed when it comes to payment. This was common last year but for now we are ok.

**I: Looking at the number of elderly people that do not contribute to the scheme how can government help NHIMA provide quality services to the nonpaying elderly members?**

Government needs to fund NHIMA more so that it pays for them services we give elderly members

NHIMA can partner with other organization that offer services to the elderly

**I: what effective measure can the government put in place to make sure the NHIS is sustainable despite having both contributing and noncontributing members?**

We need more NHIMA facilities we need NHIMA in clinics those are near the people like that old people will be walking to access healthcare. Government can monitor NHIMA operations making sure the monies are used for the intended purpose.

**I: thank very much, we come to end of the interview**

R: you are welcome

**END OF INTERVIEW**

**INTERVIEW GUIDE FOR NATIONAL HEALTH INSURANCE SCHEME** **MANAGEMENT IN THE KITWE DISTRICT**

**Interview ID: 21**

**Interview Date: 5^th^ JUNE 2024**

**Number of Participants: 01**

**Participant Title: PROVINCIAL COORDINATOR**

**Site: NHIMA Kitwe office (Kitwe District)**

**Transcriptionist: SAKALA LUCKY**

**I: What is your current role at NHIMA?**

R: I am the provincial coordinator for the Copperbelt Province which has Ndola and Kitwe offices.

**I: Kindly explain how NHIMA is conducting community awareness and sensitizing the community about the existence of the scheme?**

R: Every month as an organization we conduct community awareness through field trips called up country trips, during these trips we move with our field officers who conduct door to door visitation to people homes in different town here we have 10 districts Mufulira kalulushi, Masaiti Mpongwe and many others. Our field officer moves around the province to sensitize and register people on NHIMA.

Apart from that we hold market meetings road shows and social media we have a NHIMA page on Facebook where you can find all the information about NHIMA. The radio is another platform with these NHIMA information is spread across the province

**R: What strategies have you put in place to handle the issue of not having NHIMA pharmacies and private hospitals in most remote areas of the country?**

R: Here I can say Mpongwe Masaiti and Lufwanyama are considered rural areas and mostly lack several pharmacies. There are several factors we look for before we accredit a facility. We look at the capacity of the facility are they stocked, how is the hygiene do they have enough and qualified personnel. The challenge with rural areas is most of the health centers lack these requirements and at moment we prefer to accredit the district hospital as opposed to a clinic because people are referred to the hospital mostly

**I: In areas where there are no NHIMA offices how do you conduct NHIMA registration for the elderly members and where do they access the services?**

R: Informal registrations for the informal sector are ongoing and we have officers on the ground in different towns despite not having offices, online registrations still go on, one just needs a tablet to capture the NRC and the member will be register there and then. Secondly through our trips we visit various towns, and we get to register everyone across the country. Services are found at various district Hospitals, and we plan to accredit more facilities to help with the huge numbers.

**I: What are the significant achievements of the scheme regarding the health for the elderly in the district?**

R: The scheme through its senior citizen package have helped a lot of elderly who have different conditions to treat without paying anything. Different conditions such as cancer and other illnesses that come as you get older NHIMA is treating them, different expensive scans such as MRI, operations, lenses for the elderly dental services all these and many more are given for free to those that are 65 and above

**I: What measures have been put in place to improve service delivery for the elderly and ensure sustainability of the scheme?**

R: Right now, we make sure the paying members are compliant because we heavily depend on the subscriptions to support our services. We are still a young organization, and we are still learning on how we can partner with other to help us with funds.

**I: What are the main concerns you have received from the elderly as they visit various health centers for NHIS services?**

R: No medication, no facilities in the rural areas some appreciate NHIMA saying it has come to save their lives.

**I: What are the major challenges the scheme is facing as it delivers services to elderly members?**

R: Funding we depend on the paying members so more funding is needed to help the elderly package. Some facilities are abusing the services and claiming huge sum of money from NHIMA. Medication for elderly scans and operations are expensive plus the lenses we give them are also expensive the package is expensive and needs more funding

**I: What effective measure can the government put in place to make sure the NHIS is sustainable and provides quality services without difficulties?**

R: Build more NHIMA accredit more facilities in rural areas, employees more health personal to help in NHIMA public facilities, Monitor and make sure NHIMA is delivery quality services looking at monies they are getting from the contributing members.

**I: thank very much, we come to end of the interview**

R: you are welcome

**END OF INTERVIEW**

**Interview ID: 22**

**Interview Date: 5^th^ JUNE 2024**

**Number of Participants: 01**

**Participant Title: MEMBERSHIP ASSITANT MANAGER**

**Site: Kitwe office (Kitwe District)**

**Transcriptionist: SAKALA LUCKY**

**I: What is your current role at NHIMA?**

R: I am a Membership Assistant Manager; my role basically involves supervising registration assistance to make sure they register every Zambian without leaving anyone behind.

**I: Kindly explain how NHIMA is conducting community awareness and sensitizing the community about the existence of the scheme?**

R: We have social media page, adverts on both Tv and radio, apart from that we have field workers across the country who do door to door campaigns we also conduct road shows field trips just to sensitize people and participate in events such as graduations, different shows trade fairs you find as talking about NHIMA all these and many other platforms we use them to give information about NHIMA to the public.

**I: What strategies are put in place to handle the issue of not having NHIMA pharmacies and private hospitals in most remote areas of the country?**

R: For rural areas the problem we have is facilities do not meet our requirements, most of them are low standard talk about places like Masaiti Mpongwe and Lufwanyama most private facilities there are of low standards, so we end up accrediting the district hospital instead. As for now we are accrediting the district or mission hospitals that have some equipment that can help people before they are referred to big hospitals like the general and the teaching hospitals.

**I: In areas where there are no NHIMA offices how do you conduct NHIMA registration for the elderly members and where do they access the services?**

R: NHIMA registrations are done online or using tablets devices we have officers across the country they move even to rural areas to capture every as we leave no one behind. So, office or office registrations still go on.

**I: What are the significant achievements of the scheme regarding the health for the elderly in the district?**

R: We have managed to save a lot of lives through offering free services to the elderly, we have helped people plan for their emergency health expenses through insurance as illness can come even when you have no funds. We are providing good health services across the country. We have accredited big hospitals like Maina soko in Lusaka, Wusakile hospital in Kitwe these and many other facilities provide quality services which is a plus for NHIMA.

**I: What measures have been put in place to improve delivery service for the elderly and ensure sustainability of the scheme?**

R: To accredit more facilities under NHIMA and try to partner with other organizations so that we offer good services. We are calling on organizations that are responsible for health of the vulnerable in society to partner with NHIMA so that health services can be affordable to all citizens.

**I: What have been the main concerns you have received from the elderly as they visit various health centers for NHIS services?**

R: Lack of medicines at the hospitals and pharmacies. Lack needed equipment to carry out scans and some operations, especially in the rural areas. I have seen a lot of elderly men and women come to office to complain about drugs, delay in accessing lenses for their eyes and so on. These are some of the concerns we are working hard to try and resolve as an organization.

**I: What are the major challenges the scheme is facing as it delivers services to elderly members?**

R: Medication for elderly scans and operations are expensive plus the lenses we give them are also expensive the packaging is expensive and needs more funding. Some facilities are abusing the services and claiming huge sums of money from NHIMA. Funding we depend on the paying members, so more funding is needed to help the elderly package as you know senior Citizens including their spouses do not make payments and that’s a cost to the organization.

**I: What effective measure can the government put in place to make sure the NHIS is sustainable and provides quality services without difficulties?**

R: The government needs to build more facilities that we can accredit. Government can help us with more funding and monitor how we are utilizing the resources so that everyone benefits from this health Scheme.

**I: thank very much, we come to end of the interview**

R: You are welcome

**END OF INTERVIEW**
